# Risk factor analysis of the development of severe radiation pneumonitis in patients with non-small cell lung cancer treated with curative radiotherapy, with focus on underlying pulmonary disease

**DOI:** 10.1186/s12885-023-11520-y

**Published:** 2023-10-17

**Authors:** Hakyoung Kim, Jeongeun Hwang, Sun Myung Kim, Juwhan Choi, Dae Sik Yang

**Affiliations:** 1 Departments of Radiation Oncology, Korea University Guro Hospital, Korea University College of Medicine, 148, Gurodong-Ro, Guro-Gu, Seoul, 08308 Republic of Korea; 2 Department of Medical IT Engineering, College of Medical Sciences, Soonchunhyang University, Chungcheongnam-Do, Republic of Korea; 3 Department of Internal Medicine, Korea University Guro Hospital, Division of Pulmonary, Allergy, and Critical Care Medicine, Korea University College of Medicine, Seoul, Republic of Korea

**Keywords:** Lung cancer, Radiotherapy, Pulmonary disease, Radiation pneumonitis

## Abstract

**Background:**

We aim to identify the multifaceted risk factors that can affect the development of severe radiation pneumonitis (RP) in patients with non-small cell lung cancer (NSCLC) treated with curative high-dose radiotherapy with or without concurrent chemotherapy.

**Methods:**

We retrospectively reviewed the medical records of 175 patients with stage-I-III NSCLC treated with curative thoracic X-ray radiotherapy at the Korea University Guro Hospital between June 2019 and June 2022. Treatment-related complications were evaluated using the Common Terminology Criteria for Adverse Events (version 4.03).

**Results:**

The median follow-up duration was 15 months (range: 3–47 months). Idiopathic pulmonary fibrosis (IPF) as an underlying lung disease (*P* < 0.001) and clinical stage, regarded as the concurrent use of chemotherapy (*P* = 0.009), were associated with a high rate of severe RP. In multivariate analyses adjusting confounding variables, the presence of IPF as an underlying disease was significantly associated with severe RP (odds ratio [95% confidence interval] = 48.4 [9.09–347]; *P* < 0.001). In a subgroup analysis of stage-I-II NSCLC, the incidence of severe RP in the control, chronic obstructive pulmonary disease (COPD), and IPF groups was 3.2%, 4.3%, and 42.9%, respectively (*P* < 0.001). The incidence of severe RP was 15.2%, 10.7%, and 75.0% in the control, COPD, and IPF groups, respectively (*P* < 0.001) in the stage-III NSCLC group.

**Conclusions:**

This study revealed that IPF as an underlying lung disease and the concurrent use of chemotherapy are associated with a high rate of severe RP. In contrast, COPD did not increase the risk of pulmonary toxicity after receiving curative high-dose radiotherapy.

## Background

Radiation pneumonitis (RP), an inflammatory reaction in the lungs due to radiotherapy for the thoracic organs, most commonly develops within 3–4 months after finishing radiotherapy; however, it can occur up to 6 months after radiotherapy and can lead to permanent scarring of the lung tissue, known as pulmonary fibrosis. In some cases, RP alone can affect morbidity and mortality rates in patients with lung cancer.

Although studies on various factors affecting the development of severe RP have been conducted [1–5], a consensus has not yet been reached. In addition, remarkable advances in radiotherapy techniques have not been sufficiently demonstrated. Several studies have suggested that underlying lung diseases can affect the incidence of severe RP [6–15]; of which, idiopathic pulmonary fibrosis (IPF) has a higher incidence of RP than that of other diseases [6, 7, 16, 17]. It can lead to rapid disease deterioration after radiotherapy and substantially contributes to the poor prognosis of these patients. However, the effect of chronic obstructive pulmonary disease (COPD) on severe RP after curative radiotherapy has not been fully investigated [9–11].

In this context, we aim to identify the multifaceted risk factors that can affect the development of severe RP in patients with non-small cell lung cancer (NSCLC) treated with curative high-dose radiotherapy with or without concurrent chemotherapy, with focus on underlying pulmonary disease.

## Methods

### Patients

After obtaining the Institutional Review Board approval (no. 2023GR0216), we retrospectively reviewed the medical records of 175 patients with stage-I-III NSCLC treated with curative high-dose thoracic X-ray radiotherapy at Korea University Guro Hospital between June 2019 and June 2022. Patients who did not undergo a pulmonary function test (PFT) during the staging workup process or those who did not complete radiotherapy were excluded.

### Diagnostic scheme for lung cancer and underlying lung disease

Tumor assessment comprised obtaining PFT, chest radiographs, computed tomography (CT) scans of the chest and upper abdomen, whole-body 18F-fluorodeoxyglucose positron emission tomography with CT scans, and magnetic resonance scans of the brain as a routine staging work-up. The PFT included (1) forced expiratory volume in 1 s (FEV1), (2) forced vital capacity (FVC), (3) ratio of the two volumes (FEV1/ FVC), and (4) diffusing capacity of the lungs for carbon monoxide, before treatment. All diagnoses of underlying lung diseases, such as COPD and IPF, were confirmed by experienced pulmonologists (J.H.C.). Treatment-related complications were evaluated using the Common Terminology Criteria for Adverse Events (version 4.03).

### Treatment scheme

The planned total dose and fractions differed according to the location of lung lesions. Based on institutional protocol, stereotactic ablative radiation therapy (SABR) with a total dose of 60 Gy in four fractions was administered to patients with small-sized (≤ 4 cm) NSCLC and peripherally located tumors. For patients who received intensity-modulated radiation therapy (IMRT), two different dose-fractionation schedules were planned for delivering 60 Gy in 20 fractions in the radiotherapy alone group and 66/60 Gy in 30 fractions in the concurrent chemoradiotherapy group via the simultaneous integrated boost technique. According to the prescription guidelines, we delivered at least 97% of the prescribed dose to 95% of the PTV. The minimum and maximum doses to 1 cc of the PTV were 95% and 107%, respectively. The percentage of the lung volume that received ≥ 5 Gy (V5) and 20 Gy (V20) was maintained at ≤ 65% and 35%, respectively, and the mean lung dose (MLD) was ≤ 20 Gy.

### Statistical analyses

Overall survival (OS) was defined as the time from the start of radiotherapy until the date of death due to any cause or the latest documented follow-up visit. The 2-year OS rate was calculated using the Kaplan–Meier method and was compared using the log-rank test. To compare clinical characteristics according to the occurrence of severe lung toxicity, we used Chi-square or Fisher’s exact tests to assess categorical variables and used an independent-sample t-test to assess continuous variables. Multivariate generalized linear regression analyses were performed to assess associations between variables and RP outcomes. Statistical significance was set at *p* < 0.05 in two-tailed tests. Statistical analyses were performed using the IBM SPSS Statistics for Windows (version 24.0; IBM Corp., Armonk, NY, USA), and R statistics software version 4.3.1 (R Foundation for Statistical Computing, Vienna, Austria).

## Results

### Baseline characteristics

The patients’ clinical characteristics are summarized in Table 1. The median age of the study population was 76 years (range: 38–93 years). Most patients were men (76.0%) and current or ex-smokers (53.7%). Of the 175 patients, 93 (53.1%) were diagnosed with stage-I or -II cancer and the remaining 82 (46.9%) with stage-III cancer. Excluding patients with no underlying lung disease, 51 (51/175, 29.1%) were diagnosed with COPD and 15 (15/175, 8.6%) with IPF. Regarding the radiotherapy technique, 36 patients were treated with SABR (20.6%), and the remaining 139 were treated with IMRT (79.4%).

**Table 1:** Clinical characteristics of patients treated with curative radiotherapy (*N* = 175)

**Characteristics**	**Number**	**%**
Age [years; median (range)]	76 (38–93)	
Sex		
Female Male	42 133	24.0% 76.0%
Smoking Status		
Never smoker Current or Ex-smoker	81 94	46.3% 53.7%
Histology		
Squamous cell carcinoma Adenocarcinoma Others	81 88 6	46.3% 50.3% 3.4%
ECOG performance status		
0–1 2–3	152 23	86.9% 13.1%
Clinical Stage		
I-II III	93 82	53.1% 46.9%
Underlying pulmonary diseases		
None COPD IPF	109 51 15	62.3% 29.1% 8.6%
Radiotherapy technique		
Stereotactic ablative radiotherapy Intensity modulated radiotherapy	36 139	20.6% 79.4%

*Abbreviations*: *ECOG* Eastern Cooperative Oncology Group, *COPD* chronic obstructive pulmonary disease, *IPF* idiopathic pulmonary fibrosis

### Patterns of failure and survival outcomes

**Fig. 1:**
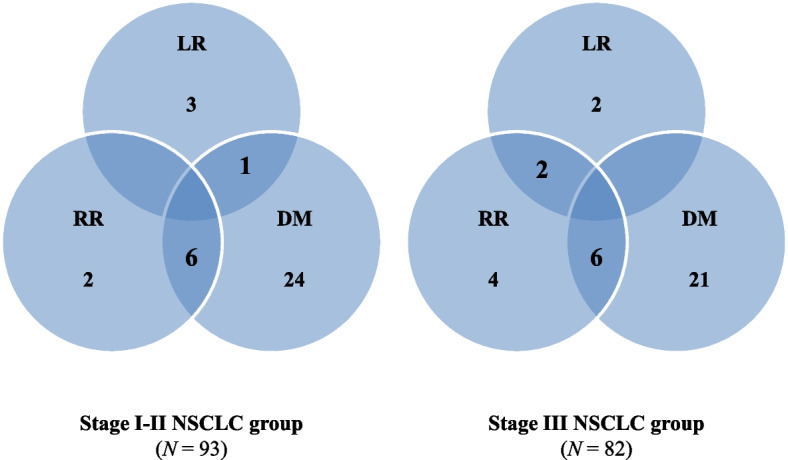
Patterns of failure in patients with non-small cell lung cancer according to the clinical stage

The median follow-up duration was 15 months (range: 3–47 months). Distant metastasis was the most common recurrent type in both stage-I and -II (31/93 patients, 33.3%) and stage-III NSCLC groups (27/82 patients, 32.9%) (Fig. 1). In terms of radiotherapy, in-field local recurrence occurred in four patients in each group. The 2-year OS rate was 72.8% in patients with stage-I or -II cancer, and 70.7% in patients with stage-III cancer.

### Treatment-related complications

The clinical characteristics, including patient, tumor, and chemo- and radiotherapy-related factors, according to the occurrence of severe RP are summarized in Table 2. No statistically significant differences were noted in sex, smoking status, histology, or pretreatment PFT values. Regarding the radiotherapy planning parameters, tumor volume and lung parameters, such as MLD, V5, V10, and V20, were similar between the two groups. However, IPF as an underlying lung disease (*P* < 0.001) and the clinical stage, based on the concurrent use of chemotherapy (*P* = 0.009), were associated with a high rate of severe RP. In multivariate generalized linear regression analysis, the presence of IPF as an underlying pulmonary disease was significantly associated with severe RP, and retained after application of backward elimination method (Table 3).

**Table 2:** Clinical characteristics according to the occurrence of severe RP (*N* = 175)

**Characteristics**	≤ **Grade 2 RP**	** ≥ Grade 3 RP**	***P* value**
Age [years; median (range)]	76 (38–93)	75 (65–87)	0.099
Sex			
Female Male	38 (90.5%) 115 (86.5%)	4 (9.5%) 18 (13.5%)	0.350
Smoking Status			
Never smoker Current or Ex-smoker	73 (90.1%) 80 (85.1%)	8 (9.9%) 14 (14.9%)	0.318
Histology			
Squamous cell carcinoma Adenocarcinoma Others	69 (85.2%) 79 (89.8%) 5 (83.3%)	12 (14.8%) 9 (10.2%) 1 (16.7%)	0.637
ECOG performance status			
0–1 2–3	131 (86.2%) 22 (95.7%)	21 (13.8%) 1 (4.3%)	0.202
Clinical Stage			
I-II III	87 (93.5%) 66 (80.5%)	6 (6.5%) 16 (19.5%)	0.009
Underlying pulmonary diseases			
None COPD IPF	100 (91.7%) 47 (92.2%) 6 (40.0%)	9 (8.3%) 4 (7.8%) 9 (60.0%)	< 0.001
Pulmonary Function Test (mean)			
FEV_1_ FEV_1_ FVC FVC FEV_1_/FVC DLco	2.1L 73.9% 3.1L 78.9% 64.7% 70.4%	2.1L 80.9% 2.9L 77.4% 71.8% 67.4%	0.469 0.082 0.213 0.093 0.533 0.274

*Abbreviations: RP* radiation pneumonitis, *ECOG* Eastern Cooperative Oncology Group, *COPD* chronic obstructive pulmonary disease, *IPF* idiopathic pulmonary fibrosis, *FEV*_*1*_ forced expiratory volume in 1 s, *FVC* forced vital capacity, *DL*_*CO*_ diffusing capacity of the lung for carbon monoxide, *CTV* clinical target volume, *MLD* mean lung dose, *V*_*D*_ percentage volume of organ receiving ≥ D Gy

**Table 3:** Associations between patient characteristics and ≥ Grade 3 radiation pneumonitis

Characteristics	≥ Grade 3 RP	*P* value	Retained
Age	4.16X10^−2^ (-2.72X10^−2^–0.120)	0.265	
Male Sex	0.931 (0.167–5.20)	0.934	
Current or Ex-smoker	0.718 (0.181–2.97)	0.636	
Histology			
Squamous cell carcinoma			
Adenocarcinoma	0.753 (0.199–2.72)	0.668	Yes
Others	0.960 (0.042–8.68)	0.974	
Clinical Stage III	3.69 (0.721–21.8)	0.129	
Underlying pulmonary diseases			
None			
COPD	1.32 (0.292–5.59)	0.706	
IPF	48.4 (9.09–347)	< 0.001*	Yes
DLco < 80%	0.264 (0.060–1.00)	0.056	Yes
Planning parameter			
Total lung_MLD Total lung_V5 Total lung_V20	1.21X10^−3^ (-2.58X10^−3^–7.01X10^−3^) -9.39X10^−3^ (-8.29X10^−2^–5.94X10^−2^) -1.27X10^−2^ (-3.49X10^−2^–9.45X10^−3^)	0.539 0.793 0.674	

*Abbreviations*: *RP* radiation pneumonitis, *COPD* chronic obstructive pulmonary disease, *IPF* idiopathic pulmonary fibrosis, *DL*_*CO*_ diffusing capacity of the lung for carbon monoxide, *MLD* mean lung dose, V_D_ percentage volume of organ receiving ≥ D Gy

^*^Odds ratios of categorical variables and βs of continuous variables and their 95% confidence intervals for Grade 3 or higher radiation pneumonitis by multivariate generalized linear regression. Variables retaining after backward elimination are marked as ‘Yes’

Regarding treatment-related lung toxicity, the incidence of severe RP was 12.6% (22/175). Specifically, the incidence of severe RP in the control, COPD, and IPF groups was 8.3% (9/109), 7.8% (4/51), and 60.0% (9/15), respectively (*P* < 0.001) (Table 4). In the subgroup analysis of stage-I-II NSCLC, the incidence of severe RP in the control, COPD, and IPF groups was 3.2%, 4.3%, and 42.9%, respectively (*P* < 0.001). The incidence of severe RP was 15.2%, 10.7%, and 75.0% in the control, COPD, and IPF groups, respectively (*P* < 0.001) in the stage-III NSCLC group. The incidence of severe RP was similar between the control and COPD groups in patients with stage-I-II and -III NSCLC. Conversely, IPF was significantly associated with a high incidence of severe RP in a cohort of patients with stage-I–III NSCLC.

**Table 4:** Radiation pneumonitis according to underlying pulmonary diseases (*N* = 175)

Characteristics	Control^a^	COPD	IPF	*P* value
All patients (*N* = 175)	109	51	15	
≥ Grade 3 RP	9 (8.3%)	4 (7.8%)	9 (60.0%)	< 0.001
Subgroup analysis with stage I-II NSCLC (*N* = 93)	63	23	7	
≥ Grade 3 RP	2 (3.2%)	1 (4.3%)	3 (42.9%)	< 0.001
Subgroup analysis with stage III NSCLC (*N* = 82)	46	28	8	
≥ Grade 3 RP	7 (15.2%)	3 (10.7%)	6 (75.0%)	< 0.001

*Abbreviations*: *RP* radiation pneumonitis, *COPD* chronic obstructive pulmonary disease, *IPF* idiopathic pulmonary fibrosis, *NSCLC* non-small cell lung cancer

^a^Non-COPD and non-IPF

## Discussion

Severe RP is one of the most common treatment-related toxicities after receiving curative high-dose radiotherapy and can affect the mortality rate of patients with lung cancer. Although previous studies on various factors affecting the occurrence of severe RP have been conducted [1–3, 18, 19], no consensus has been yet reached. In addition, the technical aspects of radiotherapy based on the latest knowledge may not be fully reflected. The following previously known risk factors are known for each parameter: (1) patient factors, including male sex, smoking history, underlying lung disease, and poor lung function; (2) tumor factors, such as tumor size and location; (3) treatment factors, such as concurrent use of chemotherapy; and (4) dose-volume histogram-based dosimetric parameters, such as V5, V20, and MLD, which are known as predictive markers for severe RP, despite some controversies.

In terms of underlying lung disease, previous studies reported that patients with interstitial lung disease (ILD) are more susceptible to developing severe RP after receiving high-dose radiotherapy [6–8, 19]. However, ILD is not a single disease, but rather a broad group of diseases that mainly lead to problems in the lung parenchyma. Some studies, confined to IPF, reported a high incidence of severe RP after radiotherapy in these patient groups [16, 17]. However, IPF itself is a rare disease, and most studies have limitations regarding the small number of patients. Moreover, the impact of COPD on the risk of severe RP development after curative radiotherapy has not been fully investigated, and there are conflicting data [9–11]. Some studies have shown that COPD is associated with a high risk of RP, whereas other studies reported that RP is relatively mild in patients with severe COPD. Regarding the radiotherapy planning parameters, there are limitations in terms of interpreting the results of existing studies, as radiotherapy treatment technology has recently switched from the previous 3-dimensional conformal radiotherapy to the more advanced IMRT or SABR [4, 5].

In this context, we aimed to identify the multifaceted prognostic factors that can affect the development of severe RP in patients with NSCLC treated with curative high-dose radiotherapy with or without concurrent chemotherapy. In the current study, clinical characteristics were divided into patient, tumor, and treatment-related factors. No statistically significant differences were detected in sex, smoking status, histology, or pretreatment PFT values. With respect to the radiotherapy planning parameters, although the tumor volume was somewhat larger in the severe RP event group, the dosimetric parameters of the IMRT and SABR treatment plans, such as MLD, V5, and V20, showed similar values between the groups. However, IPF as an underlying lung disease (*P* < 0.001) and the concurrent use of chemotherapy (*P* = 0.009) were associated with a high rate of severe RP, which is consistent with previous results. As a result of the analysis of specific underlying lung diseases, the incidence of severe RP was similar between the control and COPD groups in both the early- (3.2% vs. 4.3%) and locally advanced-stage (15.2% vs. 10.7%) NSCLC. Conversely, IPF was significantly associated with severe RP development in patients at all stages of NSCLC (early stage, 42.9%; locally advanced stage, 75.0%).

The current study had several limitations. First, it was a retrospective analysis, and there might have been selection bias. Second, the sample size was too small to show a statistically significant difference between the two groups.

## Conclusions

This study showed that IPF as an underlying lung disease and the concurrent use of chemotherapy were associated with a high rate of severe RP. Conversely, the presence of COPD did not increase the risk of pulmonary toxicity after receiving curative high-dose radiotherapy.

## Data Availability

The datasets used and/or analyzed in the current study can be obtained from the corresponding author upon reasonable request.

## Abbreviations

RP: Radiation pneumonitis
NSCLC: Non-small cell lung cancer
IPF: Idiopathic pulmonary fibrosis
COPD: Chronic obstructive pulmonary disease
PFT: Pulmonary function test
FEV1: Forced expiratory volume in 1 s
FVC: Forced vital capacity
SABR: Stereotactic ablative radiation therapy
IMRT: Intensity-modulated radiation therapy
MLD: Mean lung dose
OS: Overall survival
ILD: Interstitial lung disease
